# Brain classification reveals the right cerebellum as the best biomarker of dyslexia

**DOI:** 10.1186/1471-2202-10-67

**Published:** 2009-06-25

**Authors:** Cyril R Pernet, Jean Baptiste Poline, Jean François Demonet, Guillaume A Rousselet

**Affiliations:** 1 SFC Brain Imaging Research Centre, SINAPSE Collaboration, University of Edinburgh, Edinburgh, UK; 2 Service Hospitalier Frédéric Joliot, CEA, Orsay, France; 3 INSERM U825, Université Paul Sabatier, CHU Purpan, Toulouse, France; 4 Centre for Cognitive NeuroImaging (CCNi) & Department of Psychology, University of Glasgow, Glasgow, UK

## Abstract

**Background:**

Developmental dyslexia is a specific cognitive disorder in reading acquisition that has genetic and neurological origins. Despite histological evidence for brain differences in dyslexia, we recently demonstrated that in large cohort of subjects, no differences between control and dyslexic readers can be found at the macroscopic level (MRI voxel), because of large variances in brain local volumes. In the present study, we aimed at finding brain areas that most discriminate dyslexic from control normal readers despite the large variance across subjects. After segmenting brain grey matter, normalizing brain size and shape and modulating the voxels' content, normal readers' brains were used to build a 'typical' brain via bootstrapped confidence intervals. Each dyslexic reader's brain was then classified independently at each voxel as being within or outside the normal range. We used this simple strategy to build a brain map showing regional percentages of differences between groups. The significance of this map was then assessed using a randomization technique.

**Results:**

The right cerebellar declive and the right lentiform nucleus were the two areas that significantly differed the most between groups with 100% of the dyslexic subjects (N = 38) falling outside of the control group (N = 39) 95% confidence interval boundaries. The clinical relevance of this result was assessed by inquiring cognitive brain-based differences among dyslexic brain subgroups in comparison to normal readers' performances. The strongest difference between dyslexic subgroups was observed between subjects with lower cerebellar declive (LCD) grey matter volumes than controls and subjects with higher cerebellar declive (HCD) grey matter volumes than controls. Dyslexic subjects with LCD volumes performed worse than subjects with HCD volumes in phonologically and lexicon related tasks. Furthermore, cerebellar and lentiform grey matter volumes interacted in dyslexic subjects, so that lower and higher lentiform grey matter volumes compared to controls differently modulated the phonological and lexical performances. Best performances (observed in controls) corresponded to an optimal value of grey matter and they dropped for higher or lower volumes.

**Conclusion:**

These results provide evidence for the existence of various subtypes of dyslexia characterized by different brain phenotypes. In addition, behavioural analyses suggest that these brain phenotypes relate to different deficits of automatization of language-based processes such as grapheme/phoneme correspondence and/or rapid access to lexicon entries.

## Background

Developmental dyslexia consists of a specific and persistent failure to acquire efficient reading skills despite conventional instruction, adequate intelligence, and socio-cultural opportunity [1]. Many competing neuro-cognitive hypotheses aim to explain dyslexia. The phonological hypothesis, which is the most influential account for reading problems, postulates deficits related to the access or the manipulation of phonemic information, or both, preventing efficient learning of graphemes/phonemes correspondences that are crucial to reading; e.g. [2,3]. By contrast, the auditory processing deficit theory proposes that phonological deficits are secondary to a more basic impairment in (rapid) auditory processing [4,5]. The visual magnocellular hypothesis posits the existence of low level visual disorders related to abnormal thalamic magno-cells [6] that are involved in the processing of moving stimuli and would thus be important for reading activities related to saccadic eye movements [7]. Alternatively, the visuo-attentional hypothesis situates the impairment in the encoding of letter sequences, and this latter deficit would be dissociated from phonological deficits [8]. The cerebellar hypothesis relates dyslexia to a general learning disorder that includes a failure to automatize reading and writing skills, i.e. dyslexia is regarded as an impaired automatization of high-order sensory-motor procedures essential in reading [9,10]. Finally, the general magnocellular theory encompasses the latter four theories (basic auditory, basic visual, attentional and cerebellar) by discussing each deficit as a consequence of a general magnocellular defect [11]. According to this last theory, phonological deficits are secondary in comparison to other deficits.

This multiplicity of theories aiming to explain dyslexia reflects the heterogeneity of behavioural deficits. It is indeed becoming accepted that dyslexia is not a unique entity but might reflect different neuro-cognitive pathologies [12]. As a matter of fact, dissimilar behavioural types have been proposed for a long time. One of the first classification was proposed by Boder in 1973 [13]. Dyslexic children were distinguished on the basis of their 'sensory' deficit, i.e. they would either be classified as dysphonetic (having phonological problems), or dyseidetic (having visual problems). More recently, based on the classification of patients with acquired dyslexia, Castles and Coltheard [14] proposed to distinguish phonological from surface developmental dyslexic children. In this case, dyslexics are split into patients with assembling problems, i.e. grapheme/phoneme association, vs. patients with addressing problem, i.e. lexicon access. However, it is also recognized that developmental dyslexic children can have both assembling and addressing problems. Recent data and modelling also suggest that different subgroups can be distinguished within each subtype and that signs can be shared between these subgroups [15]. For instance, whereas patients with acquired surface dyslexia have preserved phonological abilities [14], developmental surface dyslexics present, in addition to a lexicon addressing deficit, mild phonological disorders sometimes in conjunction with a letter decoding deficit. In addition, this latter deficit (letter decoding) could also be observed in some developmental phonological dyslexics [15]. In another study [16] on adult dyslexics, Ramus et al. also found that the phonological/surface distinction does not hold for developmental dyslexia as all subjects presented phonological problems. In addition, for 70% of the subjects, phonological problems were associated with cerebellar, visual and/or auditory deficits. Other studies also pointed out the existence of different subgroups that often do not strictly follow the above mentioned theories. For instance, Heim and colleagues [17] identified, using a combined cluster/discriminant analysis, three behavioural subgroups. One subgroup had phonological deficits only (33.3% of the dyslexic subjects), another group had phonological, basic auditory and visual magnocellular deficits (35.6%), whereas the remaining group had attentional problems only (31.1%). Reid et al. [18] also observed different clusters, but using a deviance analysis: 6.6% of dyslexics had a magnocellular deficit only, another 6.6% had a cerebellar deficit only, 26.6% had a naming (fluency) deficit only, and another 20% had a phonological awareness deficit only. Other subjects (40%) presented a combination of the mentioned deficits. These and other studies [19-23] therefore suggest that one theory cannot explain all of the behavioural deficits associated with dyslexia. It is therefore not surprising that brain studies that aim to find the biological counterparts of cognitive deficits do not always agree one with the other, depending on the sample at hand and the cognitive tests used.

From a theoretical point of view, several arguments favour the idea that developmental disorders like dyslexia cannot be 'specific', i.e. cannot reflect an impairment in only one aspect of cognition like e.g. phonology [24], and this would explain why one cannot find one unique biological (area or network) counterpart of dyslexia. From the cognitive science perspective, developmental disorders should not be interpreted as an impairment in one cognitive process but rather as the endpoint of an abnormal developmental process, reflecting the interaction of deficient and compensatory processes [25]. Similarly, from a behavioural genetic perspective, genes that are involved in developmental disorders have both, specific and general effects [26] such that additional cognitive disorders or comorbidities, or both, should be observed. From an experimental point of view, recent reviews pointed out several brain areas with structural [27], or functional [12] abnormalities. Nevertheless, those reviews also agree on the heterogeneity of results across studies. In a recent paper, we [28] suggested that dyslexia has a multifocal origin in terms of brain morphology. Using a Voxel Based Morphometry (VBM) approach [29] we demonstrated that dyslexic subjects do not linearly differ from control subjects in term of local grey matter volumes. In other words, dyslexics (as a group) do not have lower or higher local grey matter volumes. By contrast, dyslexic subjects had significantly different patterns of volume variations from controls, mainly in the superior temporal suslcus, fusiform gyri and in the cerebellar declives. These patterns of volume variations correlated with pseudoword reading performances in both groups, such that dyslexics represented the lower tail of the distribution both in terms of volumes and in terms of performances. Significantly different correlations were also observed regarding phonological performances with stronger correlations in control than dyslexic subjects for the cerebellum and significant/present correlations for controls vs. absent for dyslexic subjects in the cerebrum. Finally, crisscrossed correlation patterns between dyslexic and control readers were also observed regarding the spelling performances. Our interpretation of the absence of net volume differences between groups is that, by testing a large sample of dyslexic subjects (N = 38), measures of brain volumes have too high variances (both in control and dyslexic readers), leading to accept the hypothesis of an absence of difference between groups (H0). Similarly, the possible heterogeneity of dyslexic subjects led to reject the hypothesis of a difference between groups (H1). In the present study, we further investigated this hypothesis of sample homogeneity by re-analysing the data from the same subjects tested in Pernet et al. [28]. We looked for brain areas where dyslexic subjects, as a group, were maximally different from controls. This was performed by classifying, on a single subject basis (as opposed to group comparison), each voxel of dyslexics' brains as within or outside the grey matter confidence interval observed in control subjects. Based on this brain classification, dyslexic subgroups were identified and we investigated if those subgroups showed behavioural differences. Our method contrasts with purely linear techniques (e.g. t-test) as it does not assume homogeneity of the patient population (the distribution can be e.g. bi-modal). This also contrasts with behavioural studies as subgroups are not defined by their task performances but rather defined by their brain distributions, i.e. their 'intermediate' or endophenotype [30].

Based on our previous results [28], we hypothesized that dyslexic subjects would differ maximally from controls over the left superior temporal gyrus (STG), the left and right fusiform gyri, and the left and right cerebellar declive (lobe VI). In addition, we expected only one group of dyslexic subjects over the left STG (as there was a tendency for group differences in [28]) but possibly several subgroups of dyslexics over the fusiform gyri and the cerebellum; leading to observe several brain phenotypes [30]. The new analyzes reported here revealed that dyslexics are best discriminated from controls (100% of dyslexics outside the confidence intervals) on the basis of cerebellar and lentiform nucleus volumes only. Furthermore, subgroups with higher or lower volumes in these areas differed behaviourally one from another, therefore comforting our hypothesis that dyslexia is an heterogeneous condition and, by extension, that it cannot be explained by a single 'specific' hypothesis.

## Methods

### Participants

Thirty-nine control subjects (four women; mean age 27.83 years, SD 5.75 years) and thirty-eight dyslexic subjects (four women; mean age 27.25 years, SD 7.92 years) participated in this study. All subjects were adult (i.e. above 18 years old) native French speakers, had 12 years or more of schooling corresponding for all of them to at least an A level (French baccalaureate level, passed or not). All subjects were free from any history of sensory deficits, neurological or psychiatric illness, or medical treatment. Seven subjects were left-handed and the remaining subjects were right-handed (minimal score 65% on the Edinburgh inventory test, [31]). The Toulouse local ethic committee approved the different study's protocols and all subjects gave informed written consent.

The diagnosis of developmental dyslexia was established using both inventory and testing procedures in accordance with the guidelines of the ICD-10 Classification of Mental and Behavioral Disorders. The clinical examination included a clinical interview, regular, irregular, and "loan" (foreign words that are used in French and primarily call upon addressing, lexical reading procedure), and pseudo-word reading tasks, a rapid digit-strings reading task, phonological and metaphonological tasks (syllabic deletion, phoneme deletion, spoonerisms, phonologically incongruent word search, phonological-based rime decision from visual stimuli), and spelling tasks of irregular words and pseudowords (see [32] for details). In addition, the IQ was controlled using either the full WAIS-IVR battery or the vocabulary, similarity, blocks, and assembly subtests from the WAIS-IVR. Performances (scores and RTs) of each subject were classified according to normalized scores. A participant was diagnosed as dyslexic and included into the study if his/her performance was two standard deviations below the average on at least 10 out of 21 scales of the clinical tests (scores or RTs) whereas his/her IQ was within the normal range.

### Imaging parameters

High resolution T1-weighted 3D MRI images (MPRAGE) were obtained for all subjects. Fifty-two subjects (25 dyslexic readers and 27 control readers) were scanned in a 1.5 tesla Magneton Vision Siemens scanner (FOV 300 mm, matrix 256 × 256 × 256, voxel size 1.17 mm^3^). The other twenty-five subjects (13 dyslexic readers and 12 control readers) were scanned on a 2 teslas Magneton Vision Siemens scanner (FOV 256 mm, matrix 256 × 256 × 108, voxel size 1 × 1 × 1.5 mm for 8 subjects and matrix 162 × 256 × 256, voxel size 1 mm^3 ^for the other 17 subjects).

### Brain classification

**Figure 1:**
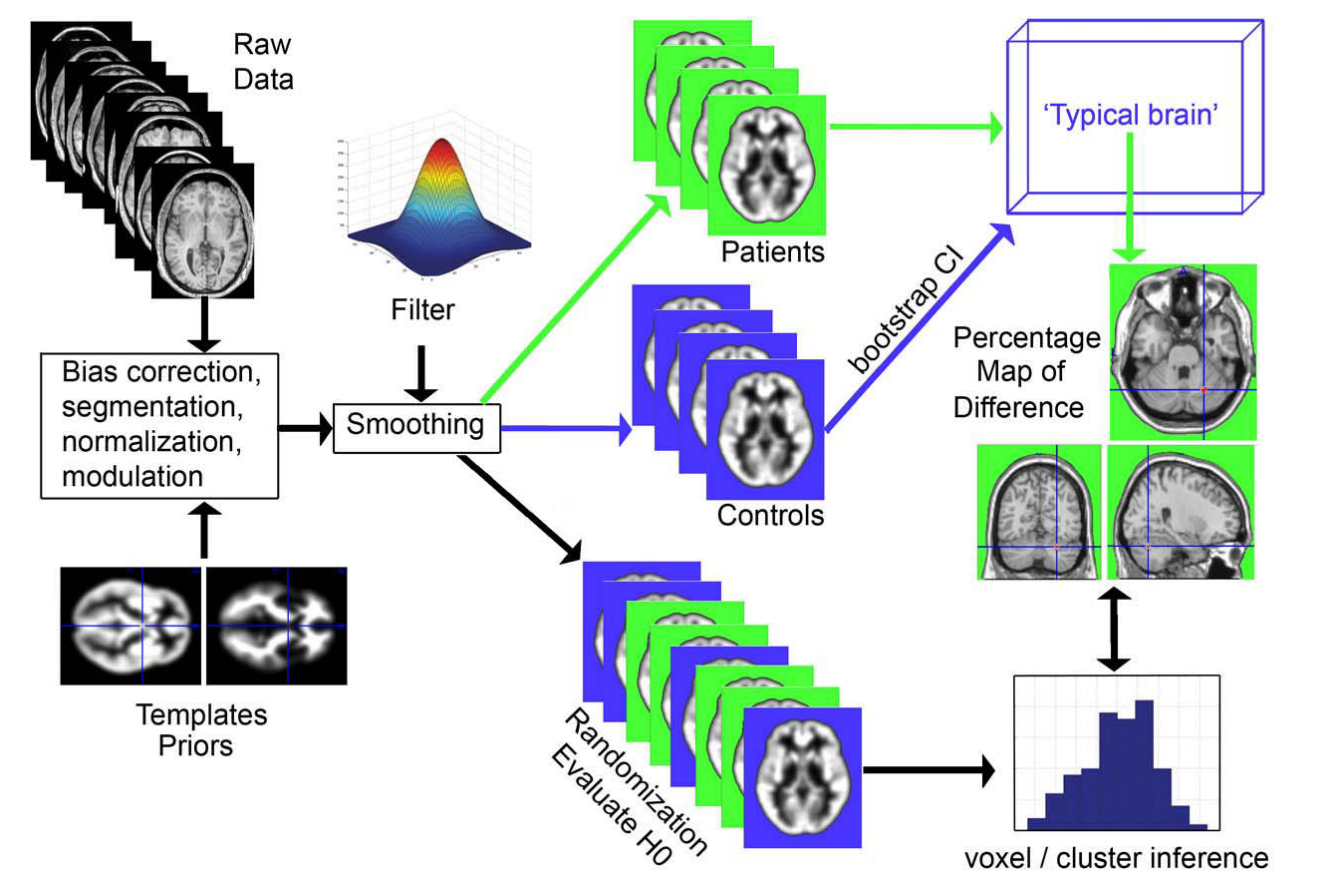
**Illustration of the processing steps from raw data to the final brain map result**. After pre-processing (bias correction, segmentation, normalization, modulation and smoothing), confidence intervals (CI) were obtained for each voxel of the brain. This allowed to construct a 'typical brain', i.e. a 5D matrix with 3 spatial dimensions, 1 dimension for the low and high bounds of the CI and the last dimension for number of CI). In this study, 5 CI were built after 3000, 3500, 4000, 4500 and 5000 bootstrap resamples. The 'typical brain' was therefore of dimensions 91*109*91*2*5 (91 voxels in x, 109 in y, 91 in z, 2 for upper/lower bound/5 for the 5 CI). Each voxel of each dyslexic subject was then classified as within or outside the 5 CIs. Results were then averaged, resulting in a percentage map of difference (PMD). A random attribution of scans to the control and dyslexic groups was used to compute the probability to find the observed values under H0, the null hypothesis according to which dyslexics and controls were sampled by chance from the same population.

Figure 1 summarizes the different processing steps of the method. First, all images were pre-processed in order to extract grey matter volume information for each voxel of the brain and spatially standardize each brain to a common space (step 1). Second, control subjects' brains were used to build 95% confidence intervals (CI) using a bootstrap procedure (step 2). Third, each voxel of each dyslexic subjects' brain was classified as being within or outside the 95% CI. Averaging across subjects resulted in a percentage map of difference (PMD) in which each voxel value reflected the percentage of dyslexic subjects falling outside the CIs, i.e. different from control subjects (step 3). Finally, the probability to find by chance the observed results was assessed by sampling subjects with replacement and assigning them randomly to the control and the dyslexic groups, and repeating steps two and three one hundred times (step 4). The average values obtained after repeating steps 2 and 3 therefore reflected the expected PMD under H0. Multiple comparison correction was performed using a maximum cluster size statistics under H0.

#### Pre-processing (step 1)

MRI data were processed in order to obtain local (voxel) grey matter volume (LGMV) information. Images were segmented into grey- and white-matter and 'other tissues', and simultaneously bias corrected and warped into a standard space (MNI) using the SPM5 (R186) toolbox running on Matlab^® ^7 (R14) software. The 'unified-segmentation' approach implemented in SPM5 [33] uses an iterative algorithm so that the optimal solution is obtained for each component. The bias correction (i.e. of spatial MR inhomogeneity) is applied such as it allows an optimal segmentation and this segmentation is optimal regarding the warping/normalization step. Both segmentation and normalization rely on the use of spatial priors (maps) indicating the probability of grey matter, white matter and other tissues. The resulting outputs were grey matter images in which the value in each voxel is the probability that a particular voxel belongs to that class. Images were then thresholded such as only probabilities superior to 0.2 were kept. As part of the process, the probability values associated with the segmentation were modulated by the local stretching and compression induced by the warps (post multiplication by the Jacobian determinant of the deformation field) such that the total content of any tissue class was the same in the warped images as it would have been in their original space, therefore reflecting the local volume [34]. Parameters used to perform the analyses were "light regularization" and 60 mm full-width at half maximum (FWHM) cutoff for bias correction, two Gaussian functions per tissue class for segmentation, 25 mm warp-field cutoff for normalization. Data were resampled at 2 mm^3 ^using a trilinear interpolation and smoothed with a 8 mm FWHM isotropic Gaussian kernel to make data more normally distributed [35].

#### Building confidence intervals (step 2)

**Figure 2:**
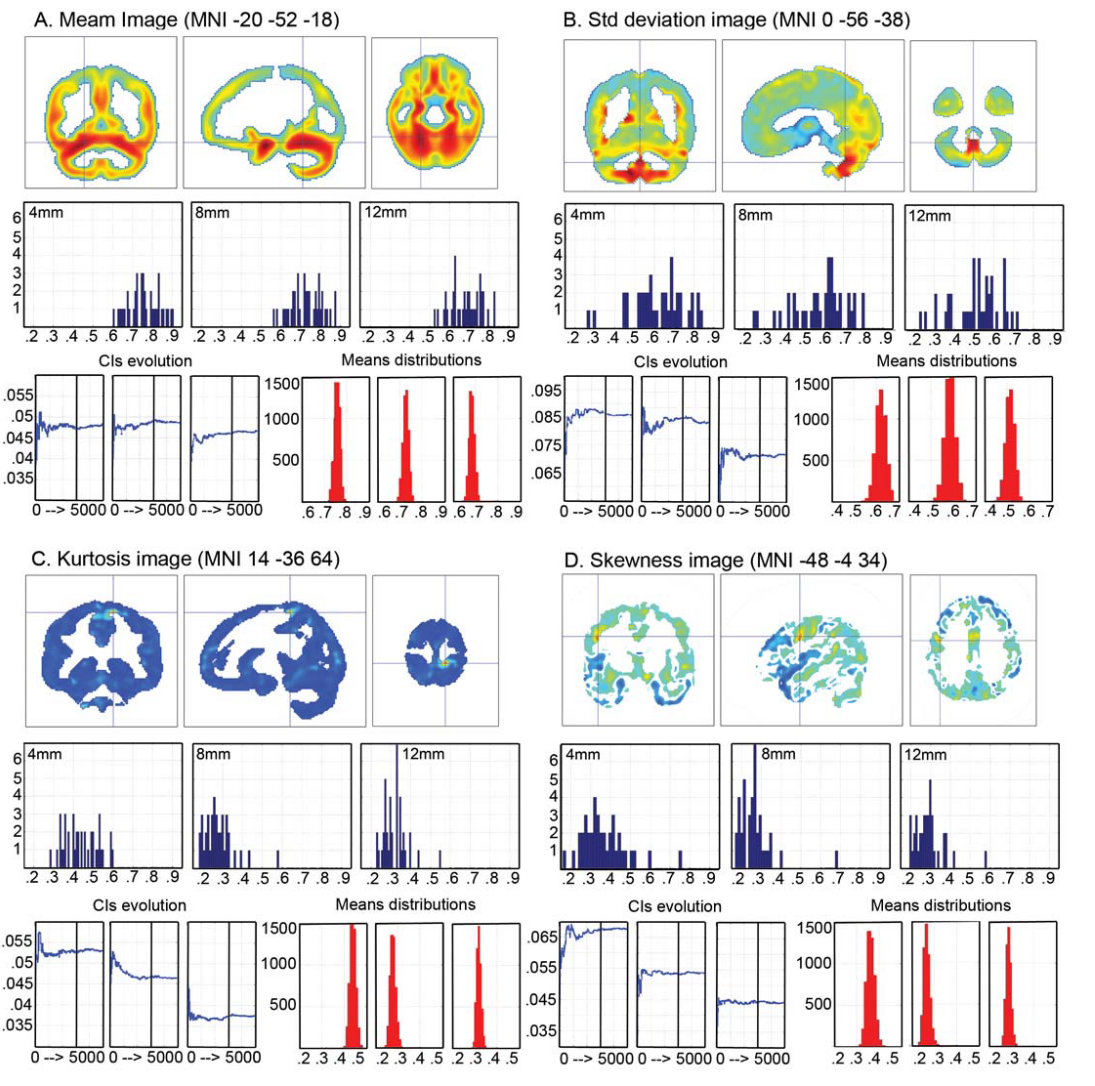
**Comparison of the bootstrapped CI for data smoothed with a 4, 8 or 12 mm FWHM isotropic Gaussian kernel**. From A to D, histograms of the grey matter values across subjects (blue histograms) are plotted for the maximum of the average image, the standard deviation image, the kurtosis image and the skewness image obtained with the 8 mm FWHM smoothing kernel. The confidence interval (CIs) evolution graphics show the size of the CIs after each bootstrap. As illustrated, CI sizes differed depending on the smoothing kernel size. However, in all cases, CI sizes converged (i.e. were stable) after 3000 resamples (vertical black line). Similarly, the final histograms of the means obtained after 5000 resamples (red histograms titled 'Means distributions'), show that the estimated data ranges differed with the smoothing kernel size but that, in all cases, it was sufficient to obtain more closely normally distributed data.

Non-normality of data is a major pitfall in confidence interval construction. To circumvent this problem, a bootstrap procedure was used. Values within each voxels and across subjects were comparable (all between 0.2 and 1) as they reflected the local volumes (see step 1). Confidence intervals were built by sampling subjects with replacement and computing means across subjects. The sampling was repeated 4,999 times (total of 5000 samples). Each time, the same subjects' sample was used for each voxel, following that subjects, but not voxels, are independent variables. The resampling procedure led to a distribution of bootstrapped estimates of the mean, averaged across subjects. The 95% percent confidence intervals were computed based on theses histograms (alpha = 0.05). This bootstrap technique relies on an estimation of H1, and tends to have more power than other robust methods like permutation tests and related bootstrap methods that evaluate the null hypothesis H0 [36]. To illustrate the advantage and robustness of the bootstrap approach over the one-sample t-test CIs, these two types of CIs are presented in the result section. Additional analyses (CI computation and classification) were also carried out on data smoothed with a 4 mm and a 12 mm FWHM isotropic Gaussian kernel in order to evaluate the robustness of the method (Fig. 2).

#### Classification (step 3)

For each dyslexic subject and for each voxel of the brain, a binary decision was made: outside (below or above), or within the CI. This procedure was repeated for 5 different CIs, computed from 3000, 3500, 4000, 4500 and 5000 resamples of control subjects. The average over the 5 classifications was computed and a percentage map of difference (PMD) created. The PMD thus reflects the percentage of dyslexics' subjects different from the theoretical normal population. Only voxels where all patients were outside the 5 CIs, i.e. showing 100% of difference, were considered here (i.e. with a priori type I error of p ~ 5 × 5% = 1%). This allowed a better control of false positive since the average classification did not depend on a particular resample. The choice of the maximum number of resamples (5000) was arbitrary. It was nevertheless sufficient since the CI size was stable after 3000 resamples (Fig. 2).

#### Test of significance (step 4)

To estimate the probability to find by chance results similar to those observed, the analysis described above was performed 100 times under the null hypothesis of no difference between groups. Each time, two groups were selected randomly, with replacement, from a data set containing controls and patients mixed together. One group was used as a control group to create the bootstrapped CIs (5000 resamples), whereas the other group was used as a patient group on which the classification was performed. The maximum likelihood estimates (mean and standard deviation) of the distributions of percentages of difference at each voxel over the 100 classifications were obtained, allowing the computation of p-values for the observed differences at each voxel. To correct for multiple comparisons, we considered cluster sizes [37]. Since we were interested in voxels showing 100% of difference, the maximum cluster size of voxels with 100% difference was obtained, over the whole brain, for each of the 100 randomizations. This gave a distribution under H0 of the cluster sizes for voxels with 100% difference, located anywhere in the brain, thus correcting for multiple testing. Confidence intervals were then build using a bootstrap procedure (5000 resamples with replacement) to compute the probabilities associated with cluster sizes.

#### Specificity and sensitivity

In addition to the estimation of the probability to find by chance results similar to those observed, we also tested the sensitivity and internal specificity of the classification results using a K-fold cross-validation. First, we computed CIs for all voxels of the whole brain from two third of the control data (step 2). Second, we classified patients and the remaining members of the control group based on the CIs computed from two third of the control data (step 3). We performed those analysis three times, by knocking out one third of the control subjects each time. Last, we compared the mean 3-fold classifications results to the initial one. This analysis tested the robustness of the patient classification results relative to the control sample used, and evaluated the specificity of the results, relative to normal subjects.

### Correlations with behaviour

Based on the brain classification results, we isolated 4 subgroups of dyslexic subjects depending on whether they had lower or higher grey matter volumes than controls in the right cerebellar declive and the right lentiform nucleus (see results). Three sets of analyses were then carried out to investigate the relationship between brain classifications and behavioural performances.

In the first set of analyses, non-linear correlations were computed to investigate the brain/behavioural mapping. Using the Kendall's Tau correlation (concordance of ranks), we investigated if brain classifications mapped directly onto behavioural deficits and classifications. Dyslexics' brain data were classified according to their position above (1) or below (-1) the CIs in the cerebellum and the lentiform nucleus (2*2 groups). Behavioural performances were classified in a similar fashion relative to each normative test (1 or -1 = 2 groups for each behavioural test). Correlations between brain and behavioural classifications were then computed for each behavioural test separately. Each dyslexic subject was also classified based on his or her behavioural scores in pseudo-word and irregular word reading, in comparison with the control group. Phonological dyslexia is a condition in which subjects show difficulties in reading pseudo-words (i.e. using grapheme to phoneme conversion rules), whereas surface dyslexia is a condition in which subjects show difficulties in reading irregular words (i.e. using their lexicon). To identify surface vs. phonological dyslexics, CIs were computed for the regression between pseudo-word and irregular word reading performances from the control subjects. Patients below the 90% CI for the regression pseudo-words against irregular words and within the interval for the regression irregular words against pseudo-words were classified as phonological dyslexics. Surface dyslexics were defined conversely and the remaining subjects were classified as mixed [14]. This resulted in 3 behavioural groups (phonological, surface or mix), and we tested how this behavioural classification correlated with the brain classification.

In a second set of analyses, a linear trend analysis was performed across groups and for each test separately (corrected p-values < .0033). Based on the mean performances in each group, linear regressions were performed between subject performances and the groups coded as a continuous regressor (1, 2, 3, 4, 5). The statistical significance was assessed via bootstrap with 600 resamples [36]. Specifically, subjects in each group were drawn randomly with replacement and linear regressions were computed each time. This allowed constructing empirical confidence intervals of the regression coefficients. If a 99.67% CI did not encompass 0, i.e. if a regression coefficient was not null with a corrected probability of .0033, the linear effect was significant. In order to assess if group assignment linearly explained all of the data, non-linear effects were also computed and tested for each resample (i.e. we compared the sum square of differences between the data and the regression model [38]).

Finally, differences between subgroups were assessed with one-way MANOVAs. Variance analyses were computed separately for the phonological, lexicon, and reading tests (3 MANOVAs on scores and 3 MANOVAs on RT, corrected p-values < .008). MANOVAs were computed using Roy's test because, for all analyzes, dependant variables were collinear (i.e. only one high eigenvalue was obtained for the sum of squares and cross product matrix [39]). Although scores and RTs tend to be correlated, analyzes were carried out separately as those two measures tend to be affected differently according to the type of dyslexia and the nature of the language material used (transparent or not [24]). For all analyzes, group assignment was entered as an independent factor (1 control group and 3 dyslexic groups: Lower Cerebellar Declive/Lower Lentiform Nucleus, Lower Cerebellar Declive/Higher Lentiform Nucleus, Higher Cerebellar Declive/Higher Lentiform Nucleus). Subjects from the Higher Cerebellar Declive/Lower Lentiform Nucleus were not taken into account because of the small sample size (N = 3). Differences between groups were assessed using post-hoc Fisher LSD tests (p < .05).

## Results

### Classification

**Figure 3:**
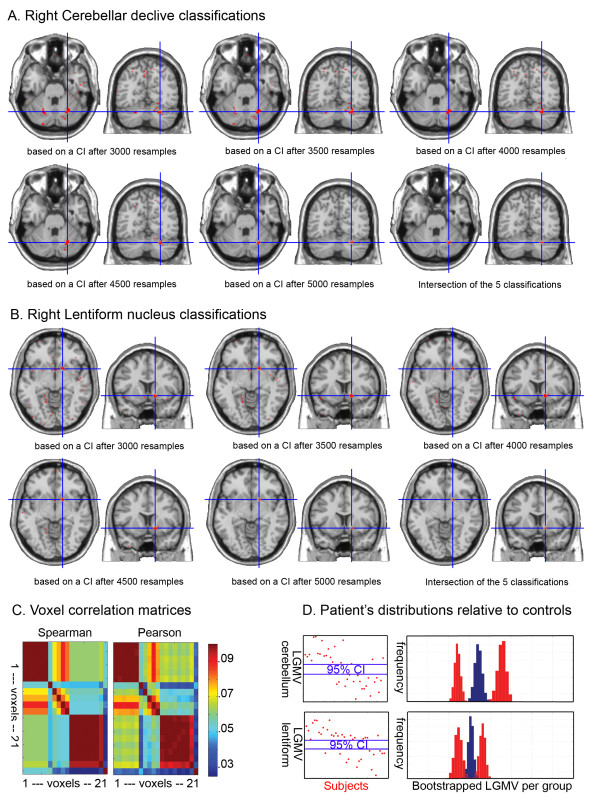
**Illustration of the results of the classification performed on the dyslexic subjects**. In A and B, the right cerebellar and lentiform nucleus clusters observed for each classification are presented (classification performed on CI computed after 3000, 3500, 4000, 4500 and 5000 resamples) as well as the final classification (the average of previous classifications). Each cluster was homogenous as illustrated in C: Among the 21 voxels showing 100% of differences between groups, two clusters can be observed with a correlation of ~1, i.e. voxels in each cluster have identical classification values (Spearman rank correlation), and identical (or nearly identical) grey matter volume values (Pearson correlations). As illustrated in D, all dyslexic subjects (red dots) were located outside the bootstrapped 95% CI (blue lines) of local grey matter volumes (LGMV). Those values are the observed value in each voxel and derived from preprocessing (step 1). Simulations (5000 resamples) of the dyslexics' distributions (red histograms) compared to the control distributions (blue histograms) show a clearer separation over the right cerebellum compared to the lentiform nucleus.

**Figure 4:**
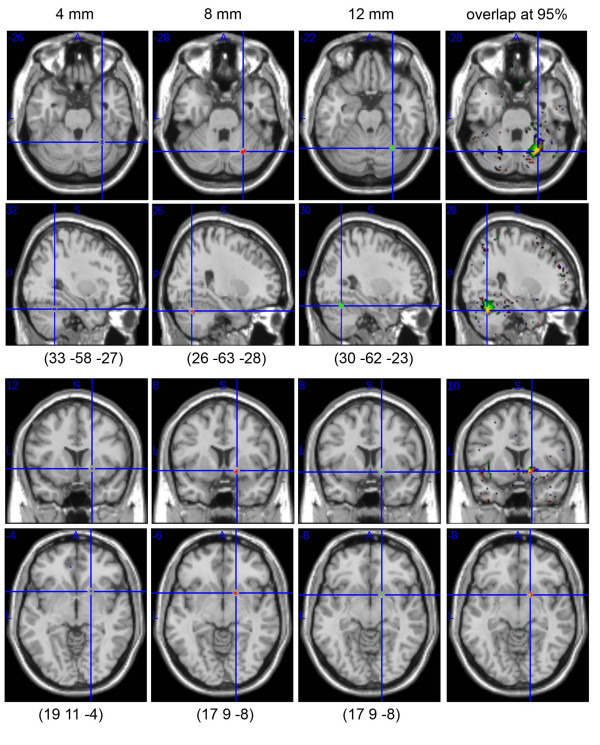
**Comparison of the dyslexic subjects' brain classifications for data smoothed with a 4, 8 or 12 mm FWHM isotropic Gaussian kernel (from left to right)**. For the three sizes, significant clusters of differences were obtained in the right cerebellar declive (corrected p values ~0; cluster size under H0 for voxels with 100% of difference = 0.36 +/- .05 at 4 mm smoothing, 0.531 +/- 0.07 at 8 mm smoothing, 1.33 +/- .09 at 12 mm smoothing). By contrast, only one cluster was observed in the right lentiform nucleus with a 8 mm smoothing kernel vs. 1 voxel only with a 4 mm or 12 mm smoothing kernel. As illustrated, the coordinates of the centres of mass (or single voxel) differed with the smoothing kernel. However, by lowering the threshold of the PMD from 100% to 95%, there is a clear overlap between the three PMD (right hand side), confirming the robustness of the results.

Based on control subjects bootstrap 95% CIs, areas that best discriminated dyslexic subjects were the right cerebellar declive (6 voxels: MNI 26 -64 -28; Fig. 3A) and the right lentiform nucleus (7 voxels: MNI 17 9 -7; Fig. 3B) with 100% of differences between control and dyslexic subjects. The bootstrap performed under H0, the null hypothesis assuming that the two groups of subjects were sampled by chance from the same population, revealed significant effects with a probability at the voxel level to find 100% of difference of p uncorrected = 0.01 for both the cerebellum and lentiform nucleus. In addition, when considering the cluster size, these two clusters had probability close to 100% (p corrected ~ 0; mean of the biggest cluster under H0 for the whole brain = 0.531 +/- 0.07). Additional analyses performed with different smoothing kernel sizes also showed clusters with 100% of differences over the right cerebellar declive (Fig. 4). In the lentiform nucleus, only 1 voxel was observed with 100% of difference for both the 4 mm and 12 mm FWHM smoothing kernel. In this case, because no cluster appeared for smaller nor for bigger smoothing kernel size, it seems likely that the difference observed with the 8 mm FWHM relates to the spatial extent of the effect (match filter theorem).

For the left superior temporal gyrus (MNI -61 -20 6) and the fusiform gyri (MNI -44/42 -65 -18), no clusters of voxels with identical classifications were observed, suggesting that those areas, although previously hypothesized as biomarkers for dyslexia, cannot discriminate controls from dyslexics. The classification values around the left superior temporal gyrus were about 84% (uncorrected p = .06 voxel level) and about 87% and 82% for the left/right fusiform gyri (uncorrected p = .05, voxel level).

### Classification vs. t-test

**Figure 5:**
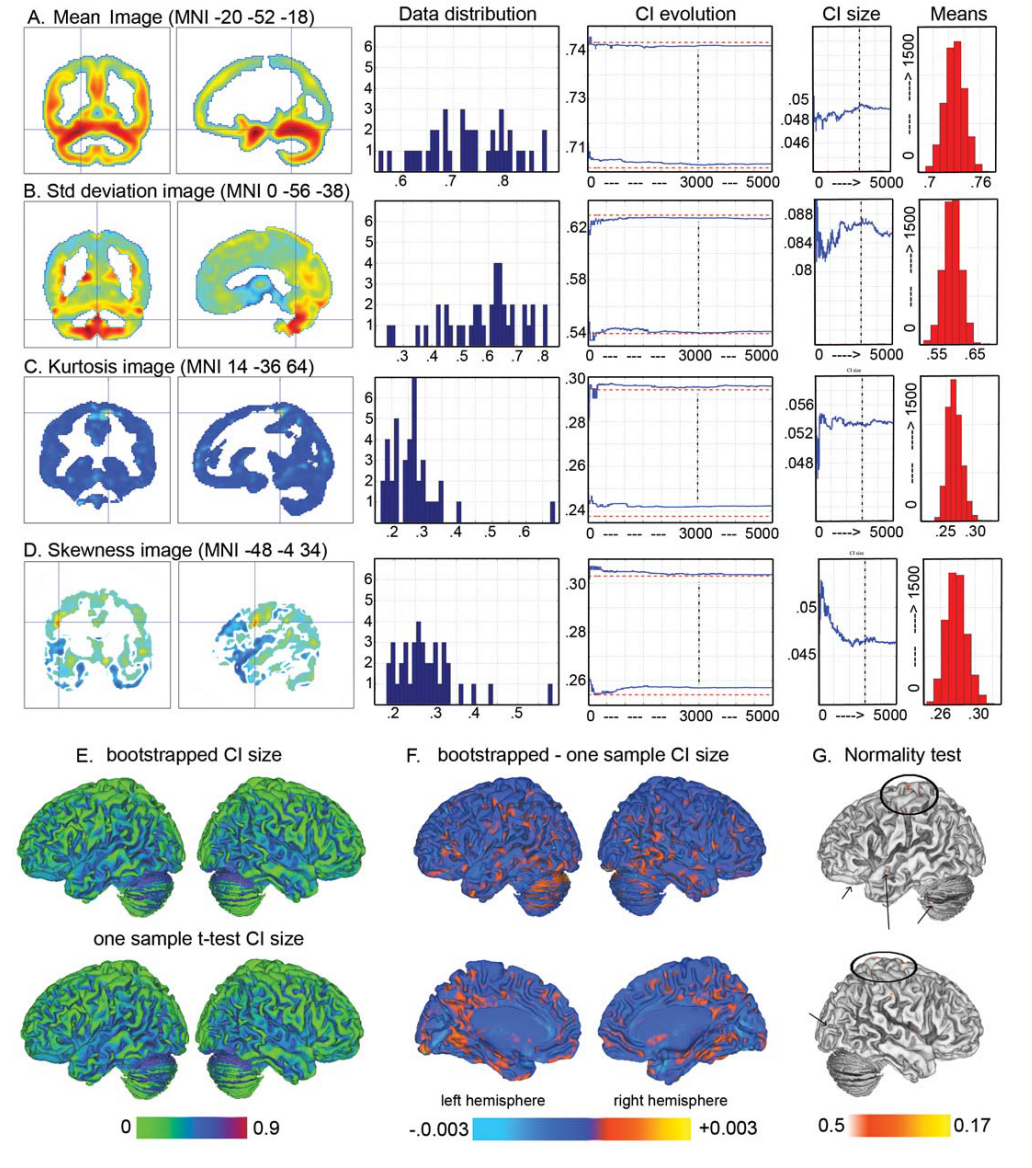
**Illustration of the advantage and robustness of the bootstrap procedure over the one-sample t-test confidence interval**. From A to D, histograms of the grey matter values across subjects are plotted for the maximum of the average image, the standard deviation image, the kurtosis image and the skewness image. As illustrated (data distribution in blue), data did not conform well (bias) to the normal distribution. This resulted in an over-estimation of the CI size using a one sample t-test (red dotted lines on the CI evolution graphics). By contrast, bootstrapped CI sizes were narrower (blue lines on the CI evolution graphics). This is illustrated over the whole brain on brain renders E and F. Overall, bootstrapped and t-test CI were similarly distributed (E) but bootstrapped CI were in general narrower (warm colours in F). The average one-sample t-test CI size was 0.0486 ± 0.0096 (min 0.0198, max 0.096, median 0.0474) vs. 0.0461 ± 0.0091 (min 0.0188, max 0.0895, median 0.045) for the 5000 resamples bootstrap CIs. This difference was statistically significant (t(251572) = 65.25 p < .00001), illustrating the advantage of the bootstrap approach, even if the majority of brain voxels have a close to Normal distributions (G – Lilliefors test > .05 – arrows and circles indicates the few 'non-normal' voxels). Graphics titled 'CI size' illustrate the evolution of the bootstrapped CI size with the number of resamples. The vertical dotted line mark the 3000^th ^resample, from which CI size tend to be stable. On the right hand side, graphics titled 'Means' show the distribution of the data means after 5000 resample (to compare with the original 'data distribution') from which the last bootstrapped CIs were obtained.

At variance with a two-sample t-test where most patients' data have to be located below *or *above the CIs (sample homogeneity) to observe differences, our method made it possible to identify areas in which patients differ from controls even if patients' data were distributed below *and *above the CIs (sample inhomogeneity – see Fig. 2A and 2B). Concretely, using a two-sample t-test, we could not observe a significant difference between controls and patients in the cerebellum (t(75) = -.48 p = .6), or in the lentiform nucleus (t(75) = -0.9 p = .37). Similarly, because the classification was performed on bootstrapped CIs that have a better control on type I error and narrower intervals than one sample t-test CIs (Fig. 5), the classification performed using the 95% one sample t-test CI failed to show any areas with 100% of differences. Using the classical approach, the classification performed on cerebellar and the lentiform nucleus voxels only reached a maximum of 94% difference.

### Specificity

Using a 3-fold cross validation, 1/3 of control subjects were classified based on 3 three different sets (folds) of CIs (computed using the other 2/3 of control subjects), leading to 3 PMD. We averaged the PMDs to test the internal specificity of the method, i.e. test if differences can be observed even if subjects are 'normal'. The average 3-fold classification showed a maximum of 95% difference (1 voxel) and the first cluster (7 voxels) appeared at 92% of difference. For the regions of interest (cerebellar declive, lentiform nucleus), clusters were observed at 85% of difference.

### Sensitivity

Using the same 3-fold cross validation, dyslexic subjects were also classified against the three different folds of CIs. The averaged PMD allowed testing the sensitivity of the method at detecting differences between groups. Over the whole brain, the average PMD revealed a maximum of 99% difference between controls and dyslexics (1 voxel). The first cluster appeared at 96% of difference in the right cerebellar culmen (MNI 8 -46 -21). Differences between groups over the right cerebellar declive (which here extended toward the right cerebellar culmen) and the right lentiform nucleus were observed at 94% of difference for both areas.

### Behavioural relevance of the brain differences

Based on the results above, each dyslexic subject was classified as belonging to one of four groups: (1) lower cerebellar declive and lower lentiform nucleus grey matter volumes than controls (LCD/LLN, N = 11, 3 females, mean age 26.8 ± 8.6); (2) lower cerebellar declive and higher lentiform nucleus grey matter volumes than controls (LCD/HLN, N = 7, 1 female, mean age 30.8 ± 10.5); (3) higher cerebellar declive and higher lentiform nucleus grey matter volumes than controls (HCD/HLN, N = 17, mean age 26.17 ± 5.87); (4) higher cerebellar declive and lower lentiform nucleus grey matter volumes than controls (HCD/LLN, N = 3, mean age 27.3 ± 11). This classification was valid regardless of the voxels considered, i.e. the same classification was observed for all cerebellar declive and lentiform nucleus voxels (inter-voxel correlation of 1 within a cluster – Fig. 3C).

#### Correlations between brain classes and behavioural classes

Kendall's rank correlation computed between each brain classification (either the cerebellum or the lentiform nucleus) and the behavioural classifications revealed only one significant effect between the classification obtained over the right cerebellum and RTs in phoneme deletion (r = -.47 p = .0039), such that dyslexic subjects with higher volumes than control subjects tended to perform well (17 subjects out of 20), whereas dyslexic subjects with lower volumes than control subjects tended to be more impaired (11 subjects out of 18). To further investigate this dissociation, dyslexic subjects were classified as phonological, surface or mixed (see method). Behaviourally, we obtained 15 phonological dyslexics, 2 surface dyslexics and the remaining 21 were classified as mixed, but this behavioural classification did not correspond to any of the brain classifications (correlations between brain classification and behavioural subtype were r = -0.16 for the cerebellum, and r = 0.15 for the lentiform nucleus).

#### Linear trends across groups

**Figure 6:**
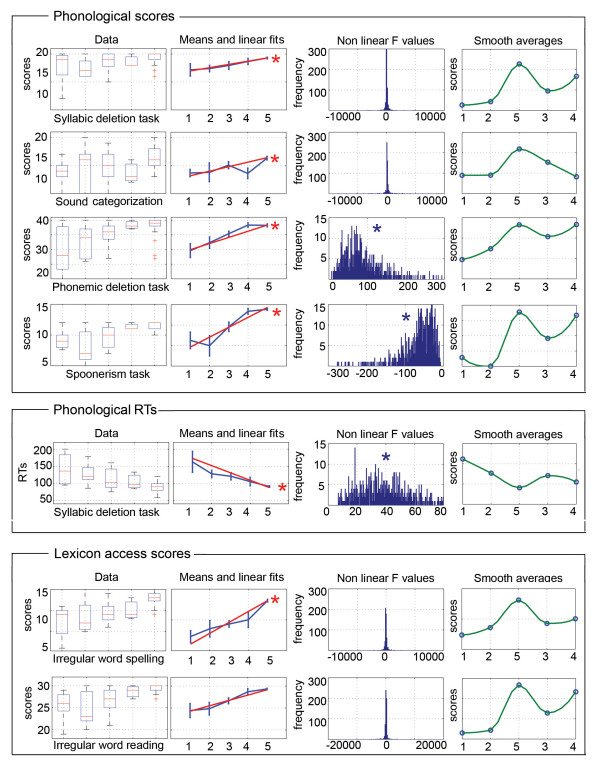
**Illustration of subjects raw performances in the boxplots titled 'Data' (the red lines indicate the median, the blue boxes extend from the upper to the lower quartile values, the whiskers show the most extreme points within 1.5 times the inter-quartile range and the red plus signs indicate outliers) and bootstrapped performances with linear adjustments (graphic lines titled 'Means and linear fits'; vertical lines show the standard deviation of the bootstrapped means and the red lines the linear fits)**. Distribution plots titled 'Non linear F values' show the distributions of F values measuring the distances between the bootstrapped data and the bootstrapped regression lines. Non central distribution suggests non linear effects; significant effects are marked with a star. For 'Data' and 'Means and linear fits' graphics, groups are ordered by increasing/decreasing mean values: (1) LCD/LLN, (2) LCD/HLN, (3) HCD/HLN, (4) HCD/LLN, (5) Controls. By contrast, the last (right end side) graphics show the bootstrapped data (blue circles) with smoothed interpolated data (in green – piecewise cubic hermite interpolating polynomial). Best scores (controls) reflected an optimal in the volume distribution: low cerebellar volumes on the left (groups 1 and 2) and high cerebellar volumes on the right (3 and 4).

**Figure 7:**
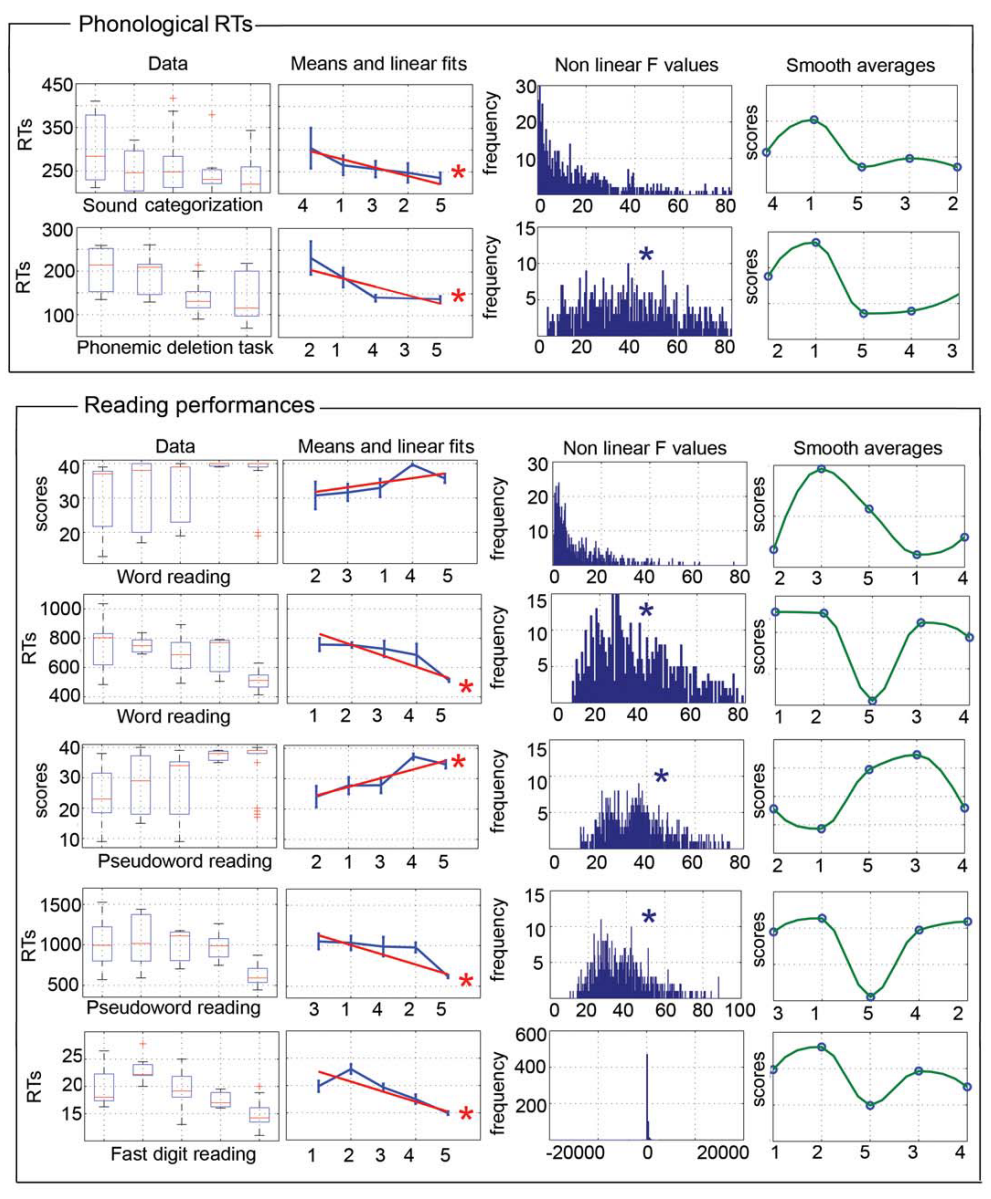
**Illustration of subjects raw performances in the boxplots titled 'Data' (the red lines indicate the median, the blue boxes extend from the upper to the lower quartile values, the whiskers show the most extreme points within 1.5 times the inter-quartile range and the red plus signs indicate outliers) and bootstrapped performances with linear adjustments (graphic lines titled 'Means and linear fits'; vertical lines show the standard deviation of the bootstrapped means and the red lines the linear fits)**. Distribution plots titled 'Non linear F values' show the distributions of F values measuring the distances between the bootstrapped data and the bootstrapped regression lines. Non central distributions suggest non linear effects; significant effects are marked with a star. For all graphics, numbers below the graphics stand for the different groups: (1) LCD/LLN, (2) LCD/HLN, (3) HCD/HLN, (4) HCD/LLN, (5) Controls. Note that although linear adjustments can be observed based on the ranking of the means, the different behavioural tests have different ranking (in contrast to the behavioural tests illustrated on the figure 6). The last (right end side) graphics show the bootstrapped data (blue circles) with smoothed interpolated data (in green – piecewise cubic hermite interpolating polynomial).

The same group ordering showed significant regression results with all the scores in phonological (syllabic deletion, phonemic deletion, sound categorization, spoonerism) and lexicon (irregular word reading and spelling) tasks. Scores across groups followed the linear ranking LCD/LLN (=1), LCD/HLN (=2), HCD/HLN (=3), HCD/LLN (=4), Controls (=5) (corrected p-values < .0033; Fig. 6). For reaction times, phonological tasks also showed significant linear trends (p < .0033). Reading performances showed more complicated results. Scores in word reading and pseudoword reading had distinct patterns. In both tasks the LCD/HLN group had the lowest performance, and the HCD/LLN group was the best dyslexic group. However, the LCD/LLN and HCD/HLN groups had reversed ranking between tasks with the LCD/LLN worse than HCD/HLN in pseudoword reading (see Table 1). These differences in ranking led to observe a significant linear trend for pseudowords, but not for word reading scores. Finally, for RTs, all tasks (word, pseudoword and digit reading) showed significant linear trends but with different orderings. Analyses (Fig. 6 and 7) revealed that linear adjustments across groups explained most of the phonological and lexicon scores (no significant non-linear effects), whereas non-linearities were observed in all RTs and in reading scores.

**Table 1:** Mean behavioural results split by brain sub-groups

	Controls	LCD/LLN	LCD/HLN	HCD/LLN	HCD/HLN
**Syllabic deletion**					
Scores	27.1 ± 5.7	17.1 ± 3.8	17.4 ± 1.6	18.6 ± 1.1	17.9 ± 3.1
RTs	90.7 ± 14.8	162.7 ± 102	127.4 ± 29.7	104.6 ± 25.3	120 ± 44.3
**Sound categorization**					
Scores	16.3 1.9	13.7 ± 2.3	13.8 ± 4.8	13.6 ± 2	15.1 ± 2.6
RTs	235.6 ± 73.6	265 ± 79.5	248.5 ± 61.7	302 ± 100	256.5 ± 76
**Phoneme deletion**					
Scores	38.2 ± 2.9	29.5 ± 8.3	32.4 ± 5.5	38.3 ± 1.5	35.5 ± 3.6
RTs	136.5 ± 49.5	188.36 ± 78	232.7 ± 106	162.6 ± 45.7	135.5 ± 34
**Spoonerism**					
Scores	11.5 ± 0.7	8.5 ± 2.9	8 ± 2.7	11.3 ± 0.5	9.8 ± 1.9
**Irregular word spelling**					
Scores	13.1 ± 1.3	7.2 ± 3.3	8.5 ± 3.7	10 ± 2.6	9.2 ± 2.1
**Loan word reading**					
Scores	29.3 ± 0.8	24.4 ± 5.5	24.8 ± 4	28.6 ± 1.2	26.6 ± 2.8
**Word reading**					
Scores	35.7 ± 8.1	33 ± 8.8	31 ± 11	39.6 ± 0.5	31.8 ± 10
RTs	512 ± 58.7	756.2 ± 159	753.2 ± 52	689 ± 158.9	726 ± 236
**Pseudoword reading**					
Scores	34.7 ± 8.2	27.8 ± 9.7	24.2 ± 9.7	37.3 ± 2	28 ± 10.5
RTs	620 ± 113.6	1033 ± 313.9	982.9 ± 172	996 ± 254.8	1056 ± 412
**Digit reading**					
RTs	14.9 ± 2.1	19.85 ± 3.56	22.9 ± 2.4	17.5 ± 1.8	19.63 ± 2.8

Table 1: For each behavioural test, the mean and standard deviation is reported for control subjects (controls) and each of the dyslexic brain subgroups (controls, lower cerebellar declive and lower lentiform nucleus grey matter volumes than controls (LCD/LLN), lower cerebellar declive and higher lentiform nucleus grey matter volumes than controls (LCD/HLN), higher cerebellar declive and lower lentiform nucleus grey matter volumes than controls (HCD/LLN), higher cerebellar declive and higher lentiform nucleus grey matter volumes than controls (HCD/HLN)).

#### Brain classification and phonological processing

MANOVAs/ANOVAs were computed to investigate differences between dyslexic sub-groups in comparison to control subjects. For this last set an analysis, the HCD/LLN group was not taken into account due to the small number of subjects (N = 3). Overall, analyses show that the subdivision of dyslexic subjects into subgroups based on their brain volumes was relevant with dyslexic subjects with lower cerebellar declive volumes showing significantly stronger phonological and lexicon access deficits than dyslexic subjects with higher cerebellar declive volumes.

In terms of accuracy, the four auditory/phonological tests showed a significant difference between groups (θ = .83 F(4,69) = 4.45 p = 1.2 10^-8^) with all 3 dyslexic subgroups having lower scores than control subjects for the syllabic deletion (F(3,70) = 3.68 p = .01), phonemic deletion (F(3,70) = 12.06 p = .000002) and spoonerism (F(3,70) = 11.7 p 1.2 10^-7^) tasks. Interestingly, for the sound categorization task, only dyslexic subjects with lower cerebellar volumes differed from controls (F(3,70) = 4.04 p = .006) with no significant difference between the HCD/HLN group and controls (p = .09), Similarly, the HCD/HLN group performed significantly better the LCD/LLN group in phonological deletion (p = .001) and the LCD/HLN in spoonerism (p = .02). In terms of processing speed, the 3 tests for which RTs were available (syllabic deletion, phonemic deletion and sound categorization) also showed significant differences between groups (θ = .59 F(3,70) = 13.77 p = 3.7 10^-7^) with stronger differences in the syllabic and phonemic deletion tasks (syllabic deletion: F(3,70) = 7.54 p = .0002; phonemic deletion: F(3,70) = 7.16 p = .0003 vs. sound categorization: F(3,70) = 0.6 p = .6). Again, the HCD/HLN group did not differ from controls in phonological deletion (p = .9) and performed faster than the LCD/LLN (p = .02) and LCD/HLN (p = .004) subgroups. Similarly for the syllabic deletion task, the HCD/HLN group was faster than the LCD/LLV subgroup (p = .02).

#### Brain classification and lexicon

Lexicon access/integrity was assessed by testing accuracy in irregular word spelling and irregular (loan) word reading. Both tasks showed a significant difference between groups (θ = 1.27 F(3,70) = 29.7 p = 1.6 10^-12^) with all dyslexic subgroups less accurate than controls (irregular word spelling F(3,70) = 29.3 p = 2.1 10^-12^; irregular word reading F(3,70) = 12.2 p = .000002). In addition, for both tasks, the HCD/HLN group performed better than the two subgroups with lower cerebellar volumes (p_max _= .04).

#### Brain classification and reading performances

The behavioural relevance of the brain classifications in terms of reading was assed by analysing data from the words, pseudowords, and digit reading tasks, but no major differences between dyslexic subgroups were observed. Accuracy in word and pseudoword reading showed significant differences between groups (θ = .36 F(3,70) = 8.56 p = .00006) with a main effect (i.e. controls > all dyslexic subgroups) observed in the pseudoword reading task only (word reading: F(3,70) = 1.1 p = .3; pseudoword reading F(3,70) = 4.4 p = .006). In terms of reading speed, a similar pattern of results was observed with control subjects always faster than dyslexic subjects (θ = 1.88 F(3,70) = 43.9 p = 4.4 10^-16^; word reading: F(3,70) = 16.5 p = 3.1 10^-8^; pseudowords reading: F(3,70) = 17.1 p = 1.8 10^-8^; digit reading: F(3,70) = 30.7 p = 8.4 10^-13^). The only difference between dyslexic subgroups concerned the LCD/HLN group which was faster than the LCD/LLN (p = .01) and the HCD/HLN (p = .004) subgroups in the digit reading task.

## Discussion

As it is the case in clinical practice, a normative reference was used in this study to enquire anatomical differences in dyslexic subjects; technically, control subjects' brains were used to compute a probabilistic norm, i.e. confidence intervals and each dyslexic's brain classified accordingly. The percentage of patients that differed from the norm at each voxel of the brain was then computed. Although relatively straightforward, this method allowed us to identify two areas where 100% of dyslexic subjects were out of the normal range: the right cerebellar declive and the right lentiform nucleus. If those areas happen to be reliable candidates to identify dyslexia (see discussion on specificity below), brain measurements could be used as a complementary tool to behavioural assessment for diagnostic [40]. In these two areas, we also observed that patients' data were distributed below and above the 95% CIs, which prevented to identify abnormalities in these areas using a linear approach [28]. This method may prove particularly relevant for patient populations with developmental or psychiatric disorders where it is often difficult to bring out a clear diagnosis based on behavioural tests only. In fact, it is even possible to have patients with similar behavioural disorders, but with physiologically or genetically different origins (e.g. [41]). Thus, being able to distinguish brain subgroups, i.e. intermediate or endophenotypes [30], could be a valuable tool for therapeutics, an idea in agreement with our discovery that brain subgroups show significant behavioural differences.

### Evaluation of the method

The use of confidence intervals to estimate group differences is at the heart of statistics [36]. Possibly because relatively new and computationally intensive, few neuroimaging studies have used resampling statistic approaches and CIs. The most popular use of bootstrapping has been in diffusion tensor imaging and tractography. On 52 hits for a search on Pubmed using 'MRI' AND 'Bootstrap' as keywords (search performed the 10^th ^Nov. 2008), 12 studies (~23%) were on DTI. In most of the studies, repetition and wild bootstrap methods have been used to quantify the uncertainty of diffusion tensors and their derived parameters (for a review see [42]). Other studies investigating morphological differences between groups have used different bootstrap methods on MRI/brain parameters derived for different regions of interest (e.g. [43]) to estimate the adequate level of probability (p-value). Here, we made full use of the resampling methods to i) estimate the normal range of grey matter volumes in each voxel of the brain and ii) provide a probabilistic classification of dyslexic subjects in different brain clusters.

Overall, the detection of areas showing 100% of difference was robust to variations in random sampling, as demonstrated by the reliability of the results with 5 different confidence intervals. In addition, differences obtained using the bootstrap were more sensitive than those detected with a one sample t-test CI (maximum of 94% of difference using one-sample t-tests CIs), a result expected because bootstrapped CIs are less sensitive to outliers and robust to deviation from normality [36] (see Fig. 5). One could argue that results were very localized with only 2 small clusters (k = 6 and 7). However, although small, those clusters had a probability to be true close to 100%. In other words, after controlling for multiple comparisons, the probability to find by chance clusters of that size with a 100% probability of being associated with a significant group difference is almost null. In addition, in each of the 5 classifications, the average cluster size was bigger (~40 voxels for the cerebellum and ~20 voxels for the lentiform nucleus; Fig. 3), but the method only keeps voxels with 100% in all 5 classifications (intersection of the 5 classifications). In fact, these two areas show strong differences in a larger extend when lowering the threshold (see e.g. Fig. 4), but we focused here on voxels with 100% of difference, hence the small cluster size.

Regarding the ability to detect areas with 100% of difference, the 3-fold validation revealed that our results were slightly dependent on the data at hand, since the mean 3-fold classification failed to obtain 100% patients different from controls (maximum at 98%). Further investigation is needed to determine why the mean K-fold classification failed. It is for instance possible that the control sample was not large enough to represent accurately the population when split in 2/3 (N = 26) and therefore the K-fold CIs were biased. Another possibility is that the dyslexic group was biased due to the selection of high-achieving adult dyslexic subjects (see below for further discussion). However, simulation of the distributions of each subgroup relative to controls (Fig. 3D) suggests that the effects would still be observed for the cerebellum with a larger group of dyslexic subjects, but would be lower for the lentiform nucleus. In all cases, because the mean K-fold classification showed again the right cerebellum and right lentiform nucleus as highly different areas and because voxels showing differences clustered again in those areas and not others, one can be confident about the results. Formal testing of our findings would request to test a new group of dyslexic subjects. However, results from the cross-validation and the bootstrap simulation of the expected populations already provide a good indicator of the long-term reproducibility of the results reported here.

The specificity of the method relative to dyslexia cannot be assessed directly without testing against other patient populations. Yet, the discovery of the right cerebellum as a major area distinguishing dyslexic from control subjects is not completely unexpected since cerebellar abnormalities have been proposed to be at the origin of dyslexia [9,10]. The implication of the right lentiform nucleus is, by contrast, unexpected. The striatum is generally involved in motor syndromes and dopamine related cognitive disorders. Some studies have implicated the striatum in language via the cortico-striato-thalamo-cortical loops that play a critical role in sequence skill learning and increasing automaticity over practice [44]. In addition Nicolson and Fawcett [10] have put forward a neurofunctional hypothesis involving both the cerebellum and the striatum in the underpinnings of different learning disorders including dyslexia. With regards to the internal specificity of these two areas, the mean K-fold classification performed on control subjects revealed 85% of difference. This suggests that the method, although relatively sensitive, is not highly specific and further work is requested to address this issue.

### Implication for dyslexia

As suggested in the introduction, dyslexia is a condition likely to reflect dissimilar neuro-cognitive disorders and no theoretical account seems able to predict the range of anatomical deficits observed in dyslexic readers [28]. Here, we show that the right cerebellar declive and the right lentiform nucleus are the two areas that maximally differ between control and dyslexic readers. Our conclusion does not imply that there are no other areas involved in dyslexia. In fact, we previously argued for the involvment of other brain areas in dyslexia [28]. However, the right lentiform nucleus, and, and in particular the right cerebellar declive (lobe VI) are the two areas showing the strongest effects. Results regarding the involvement of the cerebellum in dyslexia are not new and different histological [45], anatomical e.g. [46-51] and functional e.g. [52-54] studies pointed out abnormalities in this region. Here, we not only demonstrate abnormalities in this area, but also that dyslexic subjects could be divided into different brain phenotypes [30,55].

It is possible that our sample of dyslexic subjects was biased as they all had at least 12 years of education. However, selecting high-achieving adult dyslexics would have only potentially decreased the difference with control subjects. Despite this potential confound, we still found strong effects and distinct brain phenotypes. In addition, our samples compare well with other studies (e.g. [4,16,18-21,32,46,50,51,53]), which also worked with data from high-achieving adult dyslexic subjects.The brain variability observed among our dyslexic subjects tied up with the range of behavioural deficits observed in dyslexia (see introduction) and with genetic studies that have revealed multiple loci for chromosomal abnormalities (in particular on chromosomes 16 and 6, but also 2, 3 and 18 – [56-60]). Thus, the multifactorial and polygenic nature of developmental dyslexia strongly suggests the existence of various subtypes of patients, which could be reflected in our study by the combination of lower or higher grey matter volumes compared to controls. Furthermore, the distinction of 4 brain phenotypes of dyslexic subjects proved itself relevant because those groups differed from controls and one from each other in terms of behavioural performances. Noteworthy, none of the brain classifications, based on the cerebellum, lentiform nucleus or combined areas, followed the surface/phonological distinction. This result is however not surprising because previous data driven approaches based on behaviour did not either found groups that follow this distinction (see introduction and [16-23]). Thus, the lack of concordance between brain and behavioural classifications highlights the need for a better delineation of behavioural deficits in order to better distinguish subtypes of dyslexic readers. Finally, the linearity observed in 8 out of the 14 tests (Fig. 6 and 7) suggests that variations in Gaussian distributed brain volumes can lead to symmetrical behavioural variations, in which normal readers have the best behavioural performances associated with optimal (middle range) volumes, and dyslexic patients fall on either side of the distribution.

As mentioned earlier, the discovery of the right cerebellar declive as an area that distinguishes dyslexic from control readers could fit with the cerebellar deficit hypothesis [9,10]. In fact, the co- occurrence of the right cerebellum and the right lentiform nucleus suggests automatization deficits as a common trait across dyslexic subjects. Doyon and co-workers proposed a neural model for motor learning (see [61] for a review), in which the cerebellum is involved in early sequence learning and the basal ganglia in the automatization of motor sequences. Anatomical abnormalities in both areas therefore suggest that dyslexics present a default in motor sequence learning (cerebellum abnormality), which in turn would lead to a lack of automatization (lentiform abnormality). Although subjects have not been specifically tested for motor deficits in this study, none of them exhibited or reported motor problems during the clinical examination. In addition, because anatomical abnormalities (in particular in the cerebellum) strongly correlated with linguistic deficits; it seems plausible that cerebellar and lentiform abnormalities lead to language, not motor problems. In fact, according to Nicolson and colleagues [10], procedural learning difficulties could either appear in the language system (dyslexia), or the motor system (dyspraxia) or both. This idea is in keeping with recent reviews of language literature which highlight the cerebellum as a major locus for many linguistic tasks [62,63], and more generally in cognitive processing [64,65]. In fact, the cerebellum cluster we observed here (MNI 26 -64 -28) falls exactly within the main cerebellar/language cluster identified in a recent meta-analysis on the cerebellum [65]. Similarly, whilst right declive volumes could be used to separate dyslexic subjects into groups having strong phonological and lexicon differences, this same area has also been shown to be involved in speech perception, and more precisely in the perception of sequential inter-syllabic durations [66], i.e. phonological perception. Thus, following the idea that the cerebellum is involved in sequence learning and automatization [10,51], but that the right cerebellar declive supports language processing, we propose that cerebellar and lentiform abnormalities in dyslexia reflect specific, linguistic and reading automatization impairments [67].

## Conclusion

Using the clinician's approach, we compared each dyslexic subject to a probabilistic norm. At variance with the clinician's approach, this was performed, not for one or several behavioural measures, but for each voxel of the brain. This new method allowed us to construct a percentage map of difference that showed that 100% of dyslexic subjects differed from controls in the right cerebellar declive and the right lentiform nucleus. Further investigations showed that different brain phenotypes could be distinguished based on the cerebral volumes in those two regions. Furthermore, we demonstrated that these brain phenotypes are characterized by significantly different behavioural performances. Overall, our analyzes demonstrate that dyslexia is an heterogeneous condition (different brain phenotypes) with marked cerebral differences in the right cerebellum and lentiform nucleus. They also suggest a general, rather than specific, common deficit in linguistic automatization for all dyslexic readers.

## Authors' contributions

CRP and JFD collected the data. CRP conducted the analyses and wrote the manuscript. JBP and GAR helped analyzing the data. JFD, JBP and GAR helped revise the manuscript. All authors read and approved the final manuscript.
